# Optical Imaging of Disseminated Leukemia Models in Mice with Near-Infrared Probe Conjugated to a Monoclonal Antibody

**DOI:** 10.1371/journal.pone.0030690

**Published:** 2012-01-27

**Authors:** Sabrina Pesnel, Arnaud Pillon, Laurent Créancier, Stéphanie Lerondel, Alain Le Pape, Christian Recher, Cécile Demur, Nicolas Guilbaud, Anna Kruczynski

**Affiliations:** 1 Centre de Recherche en Oncologie Expérimentale, Institut de Recherche Pierre Fabre, Toulouse, France; 2 UPS n 44 TAAM – CIPA, CNRS, Orléans, France; 3 INSERM U618, Tours, France; 4 Service d'Hématologie, Hôpital Purpan, CHU de Toulouse, Toulouse, France; 5 INSERM U563, Centre de Physiopathologie, CHU Purpan, Toulouse, France; Institut Jacques Monod, France

## Abstract

**Background:**

The assessment of anticancer agents to treat leukemia needs to have animal models closer to the human pathology such as implantation in immunodeficient mice of leukemic cells from patient samples. A sensitive and early detection of tumor cells in these orthotopic models is a prerequisite for monitoring engraftment of leukemic cells and their dissemination in mice. Therefore, we developed a fluorescent antibody based strategy to detect leukemic foci in mice bearing patient-derived leukemic cells using fluorescence reflectance imaging (FRI) to determine when to start treatments with novel antitumor agents.

**Methods:**

Two mAbs against the CD44 human myeloid marker or the CD45 human leukocyte marker were labeled with Alexa Fluor 750 and administered to leukemia-bearing mice after having verified the immunoreactivity *in vitro*. Bioluminescent leukemic cells (HL60-Luc) were used to compare the colocalization of the fluorescent mAb with these cells. The impact of the labeled antibodies on disease progression was further determined. Finally, the fluorescent hCD45 mAb was tested in mice engrafted with human leukemic cells.

**Results:**

The probe labeling did not modify the immunoreactivity of the mAbs. There was a satisfactory correlation between bioluminescence imaging (BLI) and FRI and low doses of mAb were sufficient to detect leukemic foci. However, anti-hCD44 mAb had a strong impact on the tumor proliferation contrary to anti-hCD45 mAb. The use of anti-hCD45 mAb allowed the detection of leukemic patient cells engrafted onto NOD/SCID mice.

**Conclusions:**

A mAb labeled with a near infrared fluorochrome is useful to detect leukemic foci in disseminated models provided that its potential impact on tumor proliferation has been thoroughly documented.

## Introduction

Acute myeloid leukemia (AML) is a heterogeneous disorder of hematopoietic progenitor cells involving a block in differentiation. This incomplete maturation of leukemic blasts is usually associated with extensive cell proliferation, resulting in cell accumulation in bone marrow, peripheral blood, and eventually at ectopic locations like in the liver, in the spleen and in the central nervous system. Preclinical models that effectively recapitulate human disease are critical for expanding our knowledge of cancer biology and response to therapeutic agents. In order to develop more relevant tumor models, we implanted leukemic cells issued directly from patient samples intravenously in immunodeficient mice. However, the sensitive and early detection of leukemic cells in these orthotopic models is a prerequisite for monitoring engraftment of leukemic cells and their dissemination in mice. Two methods are currently used to detect human leukemia development in mouse models: flow cytometry analysis and human DNA assessment using Polymerase Chain Reaction (PCR) on blood samples. These methods allow the detection of circulating leukemic cells but they do not allow to access to the location of the tumor foci. One way to obtain this information is to use an imaging modality. Due to the low quantity of patient leukemic cells available and to avoid modifying tumor cell properties, cells could not be genetically modified to express luciferase gene or fluorescent protein gene which would enable monitoring the disease with direct *in vivo* imaging of bioluminescent [1] or fluorescent tumor cells [2]. So, an alternative method consisted of designing a monoclonal antibody (mAb) conjugated to a near-infrared probe with better tissue penetration and less autofluorescence than a visible fluorophore [3], [4]. Leukemic cells were characterized by different identification markers, used in flow cytometry, such as the CD44 myeloid and the CD45 leukocyte markers. CD44 is expressed by leukocytes, erythrocytes, epithelial cells and weakly by platelets; it has a functional role in cell migration, lymphocyte homing and adhesion during hematopoiesis and lymphocyte activation [5], [6]. CD45 or leukocyte common antigen is present on all human leukocytes [7] and on the surface of 85% to 95% of both B-cell lymphoma and leukemic cells [8]. So we tested mouse anti-human mAbs against these two markers. Anti-CD45 mAb is already used in clinic for immunoradiotherapy to target a radioisotope to tumor cells [9]–[11].

Therefore, in order to establish a diagnostic tool to detect leukemic foci and to perform staging of the disease in mouse models, we generated two fluorescent antibodies. We first validated this methodology by using an *in vivo* model of luminescent human leukemia HL60-Luc which expresses both hCD44 and hCD45 to compare bioluminescent imaging (BLI, tumor cells) and fluorescence reflectance imaging (FRI, mAb). We then applied this method on leukemic cells from patient samples *in vivo*.

## Methods

### Reagents

Antibody labeling kit containing AF750 dye (SAIVI^TM^ Rapid Antibody Labeling kits) was purchased from Invitrogen (Cergy-Pontoise, France). The following mAbs were used: purified NA/LE mouse IgG1 anti-human CD44, clone 515 and NA/LE mouse IgG1 anti-human CD45, clone HI30 (BD Biosciences, Le Pont de Claix, France). This anti-human CD44 mAb recognizes all the isoforms of the hCD44 cell surface glycoprotein and the HI30 CD45 mAb reacts with all isoforms of human CD45.

### Labeling of antibodies

The antibodies were labeled according to the manufacturer's instructions. The protein concentration and the degree of labeling were calculated with the absorptions at 280nm (protein) and 752nm (dye).

### Cells

The human leukemia cell line HL60 purchased from the ATCC (Manassas, VA, USA) was stably transfected with the firefly luciferase reporter gene and clonally selected to generate the HL60-Luc cell line [12]. This cell line was grown in RPMI 1640 supplemented with 20% foetal calf serum, 100 units/ml streptomycin, 100 units/ml penicillin and 1.25 µg/ml fungizone. Immediately before implantation in NOD/SCID mice, cells were centrifugated and adjusted to a concentration of 25×10^6^ cells per ml in PBS for intravenous injection. Cells were maintained in a 5% CO_2_ humidified atmosphere at 37°C. Materials for cell culture were obtained from Gibco (Cergy-Pontoise, France). The human colorectal carcinoma cell line HCT116-Luc was purchased from Caliper (USA). This cell line was grown in MEM supplemented with 20% foetal calf serum, glutamine 100 units/ml streptomycin and 100 units/ml penicillin. Cells were tested negative for mycoplasma or bacterial contamination by an independent laboratory (Clean-cells S.A., Boufféré, France).

The studies were approved by the scientific committee of the Centre de Ressources Biologiques des Hémopathies Malignes (INSERM Midi-Pyrénées). For human samples, written consent was obtained and all the data were analysed anonymously. Diagnosis was made using cytomorphology, cytogenetics and leucocyte antigen expression and evaluated according to the French-American-British (FAB) classification. The characteristics of this sample were: FAB5, normal karyotype, FLT-ITD NPM mutated. The phenotypic characteristics of this sample before grafting into NOD/SCID mice were: 92.1% of CD45+, 89.8% of CD33+, 5.1% of CD34+, 11.3% of CD38+ and 10.1% of CD123+, and after grafting were: 85.3% of CD45+, 89.6% of CD33+, 1.7% of CD34+, 62.9% of CD38+ and 1.8% of CD123+.

### Cell binding

Cell binding studies with the AF750-mAb at a single concentration of mAb (5 µg/ml) were carried out in triplicate using HL60-Luc cells (1×10^6^ cells per experiment) and HCT116-Luc cells (negative control, does not express CD45, 1×10^6^ cells per experiment). Cells were incubated in the presence of the AF750-mAb for 2h at room temperature. For control or nonspecific uptake experiments the cells were first saturated by incubating with an excess of non-fluorescent mAb (100 µg/ml) for 0.5 h. The total fluorescence was measured with an IVIS Lumina II (Caliper, USA) after which samples were centrifuged and washed twice in PBS. Student's *t* test (p<0.05) was used to determine statistical differences in the cell binding of the fluorescent mAbs.

### Animal tumor model

Homozygous female NonObese Diabetic/Severe Combined ImmunoDefiency (NOD/SCID) mice (NOD.CB17-*Prkdc^scid^*/J, Charles River Laboratories, Saint-Germain-sur-L'Arbresle, France), were used for implantation of human AML cells. These mice have a severe combined immunodeficiency affecting T- and B-lymphocyte development and a natural killer cell functional deficit, so they are adapted to the engraftment of human cells. Animals were handled and cared for in accordance with the Guide for the Care and Use of Laboratory Animals (National Research Council, 1996) and the European Directive EEC/86/609, under the supervision of authorized investigatorsAll mice received sublethal irradiation 24 hours before either i.v. transplantation with either 5×10^6^ HL60-Luc cells or 2×10^6^ patient AML cells. The experiments were performed 7 to 30 days after leukemia cell injection.

### Bioluminescence imaging

Animals were peritoneally injected with 125mg/kg luciferin potassium salt (Caliper Life Sciences, Roissy, France) before undergoing anesthesia with 3% isoflurane (Aerrane®, Maurepas, France) in air in an anesthesia induction box. Then 2% isoflurane in air/O_2_ was continuously delivered via a nose cone system in the dark box of a high sensitivity CCD camera cooled to −90°C (IVIS Lumina II, Caliper, USA). BLI was performed 10 minutes after substrate injection. Acquisition setting (binning and duration) were set up depending upon tumor activity at the time of acquisition.

### Fluorescence imaging

Fluorescent mAb was injected via the tail vein into HL60-Luc tumor-bearing mice. After 24h, mice were anesthetized then fluorescent images were obtained (after fur removal) with the Lumina II (Caliper) using one filter set (excitation: 745nm, emission: 810nm). Acquisition setting (binning and duration) were set up depending upon tumor activity at the time of acquisition.

### Determination of the minimal fluorescent monoclonal antibodies dose required and effects of fluorescent monoclonal antibodies on *in vivo* leukemia proliferation

Several doses of mAbs, between 1 and 10 µg were intravenously injected to leukemia-bearing mice. Mice were imaged 24 and 48h after using first BLI to locate the tumor foci, and then using FRI. The images were then compared to determine if all bioluminescent foci were revealed with the fluorescent mAbs and the colocalization was assessed by the calculation of Pearson correlation coefficient (ImageJ software).

In a second experiment, mice (n = 5 for anti-hCD44 mAb, n = 6 for anti-hCD45 mAb) received one injection per week for three weeks of the minimal dose of the fluorescent mAb or PBS (control group) to determine the effects of the mAb on leukemia growth. Leukemia progression was monitored using BLI and life span was recorded. Survival distribution of treated and control groups of HL60-Luc tumor-bearing mice were statistically compared using the Log-rank test. Leukemia growth inhibition was calculated from BLI data, as the ratio of the median bioluminescent signal of mAb-treated versus control groups: T/C (%)  =  (median bioluminescent signal of mAb-treated group on day X / median bioluminescent signal of control group on day X) x 100.

### Use of the fluorescent AF750 anti-hCD45 monoclonal antibody to detect leukemic foci in an experimental model of patient acute myeloid leukemia sample

5 µg AF750 anti-hCD45 mAb have been intravenously injected to patient AML cells-bearing mice. After 24h, mice were imaged using FRI. Fluorescent bones were removed to assess the number of leukemic cells present in mouse bone marrow with flow cytometry and an immunohistochemical analysis was performed to detect human CD45+ cells in order to confirm that the fluorescent signals correspond to tumor foci. The fluorescent organs were also removed to perform immunohistochemical analysis.

### Immunohistochemical and flow cytometry analyses

Human CD45+ cells were detected by immunohistochemistry in formalin/paraffin-embedded sections of bone or organs. Sections were stained using an automated system DAKO Autostainer.

Bone marrow cells were flushed from the tibia and the femur and made into single cell suspensions for analysis by flow cytometry to determine the percentage of CD45+ cells over the total number of blasts.

## Results

### *In vitro* validation

The degree of labeling calculated from the absorptions at 280nm and 752nm were 1.96 and 2.1 for the AF750 labeled anti-hCD44 mAb and anti-hCD45 mAb, respectively.

Measurement of fluorescent AF750 mAb binding to cells was done to confirm the receptor-specific targeting of the mAb labeled with AF750. The cell binding was highest with HL60-Luc (leukemia) and negligible with HCT116-Luc (colon cancer). The majority of the binding could be blocked by first incubating the cells with an excess of non-fluorescent mAb. The percent of total AF750-mAb bound to HL60-Luc cells after 2h was 61±11 and 35±7 for the AF750 labeled anti-hCD44 mAb and anti-hCD45 mAb, respectively. When the cells were first incubated with an excess of mAb to saturate the receptors, the percent of total AF750-mAb bound fell to <3% for each mAb.

### Determination of the minimal fluorescent monoclonal antibody dose

**Figure 1:**
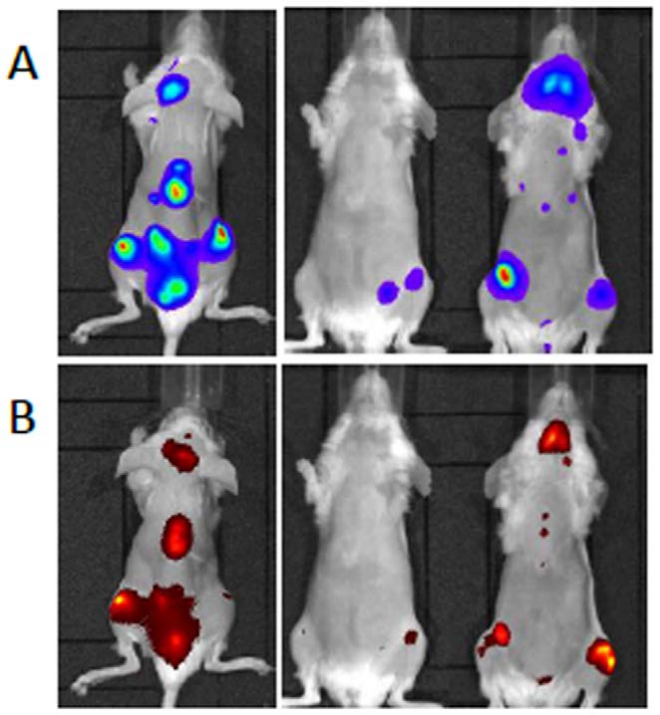
Bioluminescent (A) and fluorescent (B) of HL60-Luc tumor-bearing mice 48h post-injection of 1 µg fluorescent CD44 mAb.

zSeveral doses of mAbs, between 1 and 10 µg were intravenously injected to leukemia-bearing mice to determine the minimal fluorescent mAb dose to detect all the tumor foci. In the case of anti-hCD44 mAb, a dose of 1 µg was sufficient to detect all bioluminescent leukemic foci in the mice with a good co-localization between fluorescent and bioluminescent signals with an average Pearson correlation coefficient of 0.71 (Figure 1) and a maximal intensity obtained 48h post-injection of the fluorescent mAb.

**Figure 2:**
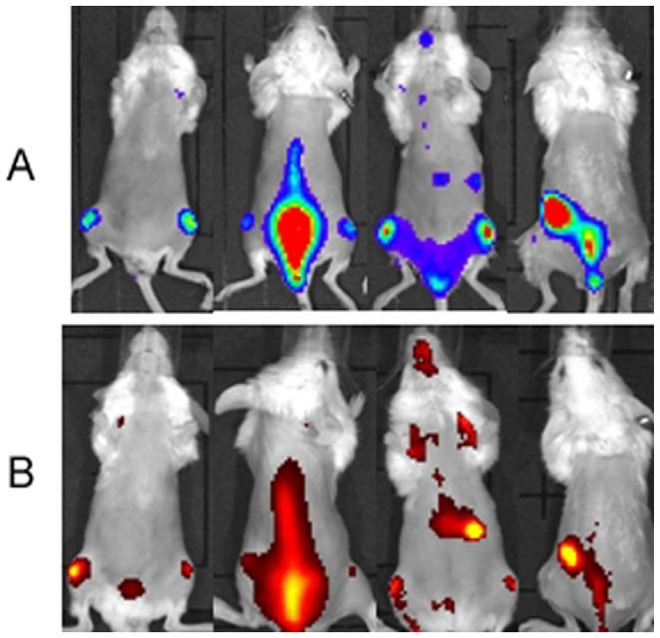
Bioluminescent (A) and fluorescent (B) images of HL60-Luc tumor-bearing mice 24h post-injection of 5 µg fluorescent CD45 mAb.

For anti-hCD45 mAb, a dose of 5 µg was required to detect all the foci with a good co-localization between fluorescent and bioluminescent signals with an average Pearson correlation coefficient of 0.65 (Figure 2) with an optimal imaging point 24h post-injection of the fluorescent mAb.

### Effects of the fluorescent monoclonal antibodies on HL60-Luc leukemia development in mice

**Figure 3:**
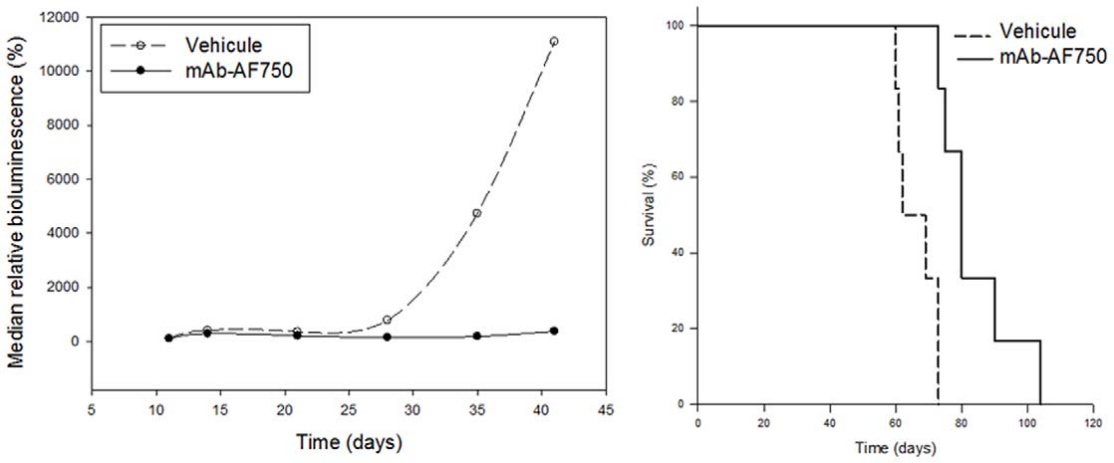
Effects of fluorescent CD44 mAb intravenously administered over three weeks on the bioluminescence signal of HL60-Luc tumor-bearing mice (A) and on life span (B).

One week after the first injection of 1 µg fluorescent anti-hCD44 mAb, BLI showed a decrease of HL60-Luc leukemia progression and the maximal leukemia growth inhibition achieved was 98.8% (T/C = 1.2%) on day 49 (Figure 3a). Furthermore, the bioluminescent signal of all the foci decreased whatever their location in the mice. This indicated a satisfactory biodistribution of this mAb allowing an exposure of all the tumor foci. In addition, treatment with anti-hCD44 mAb resulted in a significant increase of survival of leukemia-bearing mice, as assessed by the Log-rank test (p = 0.009) (Figure 3b). The anti-hCD44 mAb had a very strong impact on this leukemia model.

**Figure 4:**
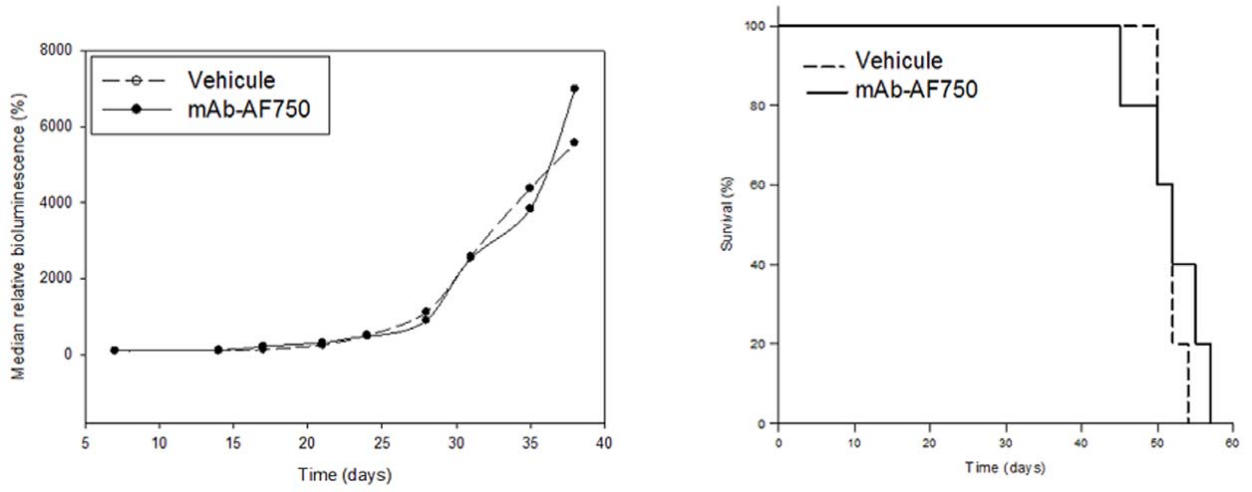
Effects of fluorescent CD45 mAb intravenously administered over three weeks on the bioluminescence signal of HL60-Luc tumor-bearing mice (A) and on life span (B).

In contrast, repeated injections of 5 µg fluorescent anti-hCD45 mAb did not modify *in vivo* leukemia progression, as assessed using BLI. Indeed, there was no significant difference on the bioluminescent signal progression between the control group and the mAb treated group (Figure 4a). Furthermore, treatment with anti-hCD45 mAb did not result in any modification of the survival of HL60-Luc-bearing mice, as assessed by the Log-rank test (p = 0.411) (Figure 4b). So, it would seem that the anti-hCD45 mAb has no impact on the leukemia progression in this model.

### Detection of patient acute myeloid leukemia cells in NOD/SCID mice using the fluorescent AF750 anti-hCD45 monoclonal antibody

**Figure 5:**
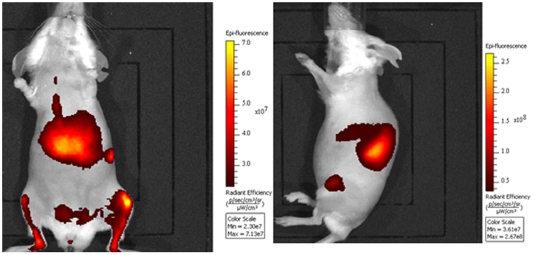
Fluorescence imaging of patient AML cells bearing mice performed 24h after intravenous injection of fluorescent CD45 mAb (ventral and lateral views).

**Figure 6:**
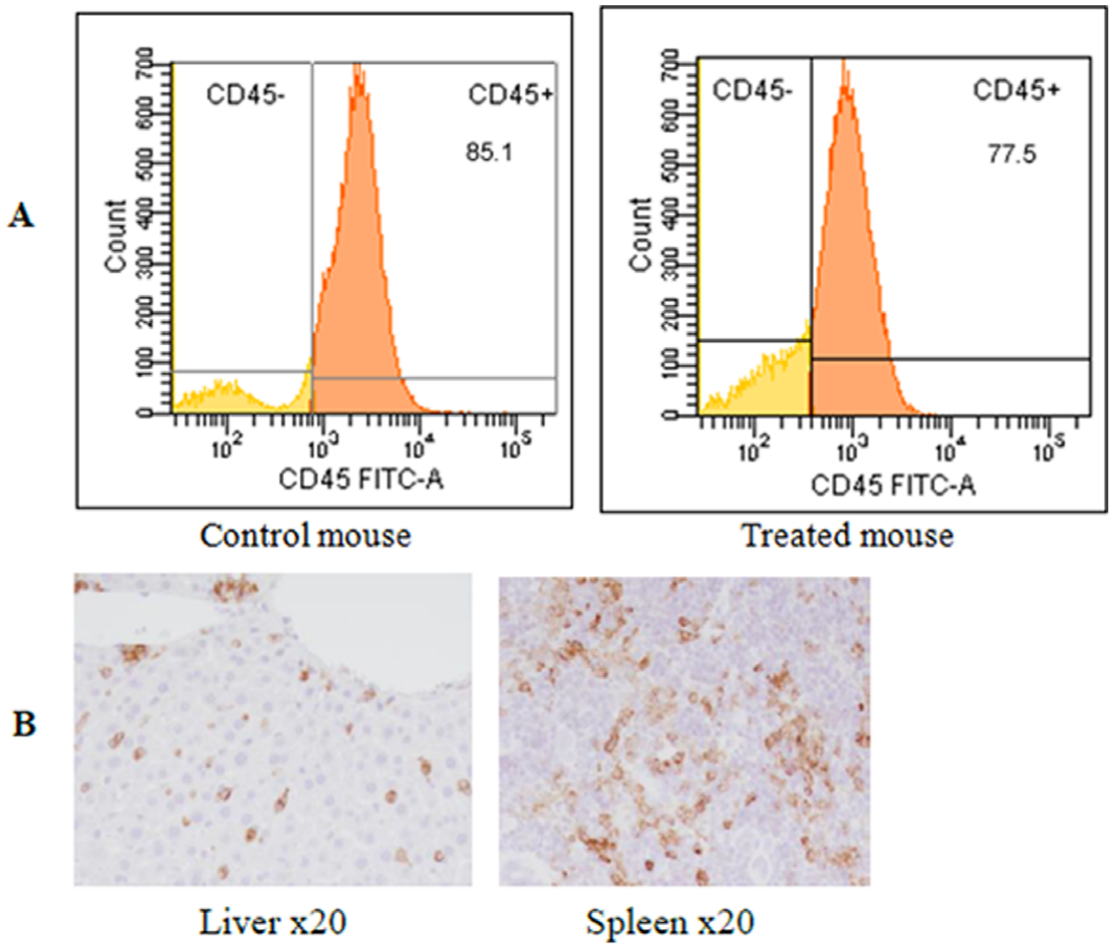
(A) Representative flow cytometric analysis of bone marrow of a mouse transplanted with leukemic cells from patient AML sample treated or not with anti-hCD45 mAb. The bone marrow is overrun by CD45+ cells in the two cases. (B) Liver and spleen CD45 immunostaining at x20.

The FRI performed 24h post-injection of the fluorescent anti-hCD45 antibody to AML-bearing mice showed several fluorescent foci in the femur, tibia, sternum spleen and liver (Figure 5). To confirm the specific fixation of the mAb on the human leukemic cells, flow cytometry and immunohistochemical analysis were performed on the organs showing a fluorescent signal. The flow cytometry analysis revealed a massive invasion of the bone marrow by hCD45+ leukemic cells (Figure 6a); 90% and 70% of the blasts present in the tibia and the femur, respectively, were hCD45+. The same results were obtained with control mice (without anti-hCD45 mAb injections). The immunohistochemical analysis of the bones, spleen and liver confirmed the presence of hCD45+ leukemic cells (Figure 6b). It showed that cancer cells had extensively invaded the bone marrow. These results revealed a satisfactory correlation between the fluorescent signal emitted by the anti-hCD45 mAb and the presence of leukemic cells in the bone marrow and other organs (spleen, liver) demonstrating efficient targeting of the fluorescent mAb in the mice.

## Discussion

Our aim was to develop a fluorescent probe for detection and staging of human leukemic foci in mice bearing patient-derived leukemic cells in order to determine when to start treatments of the mice with novel antitumor agents.

Three strategies can be envisaged to image tumor cells without genetically modifying them. The first class uses cell markers: it is possible to use lipophilic tracers such as 1,1-dioctadecyl-3,3,3,3-tetramethylindotricarbocyanine iodide (DiR) or 1,1'-dioctadecyl-3,3,3′,3′-tetramethylindotricarbocyanine (DiD) [13]. This type of tracer cannot be used to follow cells for a long time because at each cell division the number of fluorochromes presents on the cells decreases. Another option consists in labeling cells with radioisotopes (Indium 111 [14] or Copper 64 [15]) but it is not possible to image the cells for long time due to the short half-life of the isotopes. The second class uses metabolic markers such as the 18F-FluoroDeoxyGlucose (FDG) [16], [17] but they are not tumor specific. The last class uses molecular targeted probes that are imaging agents against tissue modifications associated with a pathology or a cellular type such a the CD45 which is a marker of leucocytes. This targeting can be realized *via* an antibody, a peptide (for example the RGD peptide which binds to integrins) or the ligand of a receptor. For our application, we have chosen to use an antibody specific of the human leukemic cells. Antibodies can be labeled with chelates that bind radioactive metals [18], [19]. However, radiolabeling exposes mice to ionizing radiations that can impact tumor progression in the case of repeated exams of radiosensitive tumors. Furthermore, despite high resolution and sensitivity, nuclear imaging suffers from severe constraints due to requirements of dedicated facilities and regulations about radiation use. Optical labeling overcomes these disadvantages but suffers from poor tissue penetration. Among the fluorochromes, near-infrared dyes have the greatest tissue penetration and a low autofluorescence, and so are the most used for *in vivo* imaging. We decided to use the fluorochrome Alexa fluor 750 because its emission wavelength at 780nm is best suited to reveal deep foci and with the additional advantage over Alexa fluor 680 of an absence of autofluorescence in abdomen. In cancer research, the most routinely used imaging modality to measure tumor burden is the BLI [20], [21], so we used it as a standard for comparison in this study. The use of fluorescence imaging has developed substantially in recent years due to the rapid increase in availability of fluorescent probes with varying complexity. This modality suffers from tissue absorption and scattering (more important than in BLI) making tough the quantification of the signal. However with improvements in advanced fluorescence imaging modalities, such as fluorescence molecular tomography (FMT), the sensitivity and resolution of deep foci will be sufficient to allow the absolute quantification of signal. It is in this context that this new strategy was developed.

Cell-based studies confirmed that the immunoreactivity of the antibodies was not compromised and that each of the AF750-labeled mAb could selectively bind hCD44 or hCD45 expressing cells.

All the studies were performed on the human leukemia cell line HL60 because it expresses both hCD44 and hCD45. In this model, both fluorescent anti-hCD44 and anti-hCD45 mAb achieved good co-localization (Pearson correlation coefficient >0.5) between fluorescent signal (mAb) and bioluminescent signal (leukemia cells) using low injected doses: 1 µg and 5 µg of anti-hCD44 and anti-hCD45 mAb, respectively. Increased labeling may lower the dose requirement but runs the risk of losing immunoreactivity.

However, although the required fluorescent anti-hCD44 mAb dose was very low, it induced a strong impact on leukemic cell proliferation. Indeed a marked inhibition of leukemia development was measured using BLI and the survival of HL60-Luc-bearing mice was significantly increased. An anti-leukemic activity of anti-hCD44 mAb has been reported previously but at higher doses than the ones used in the present study (3mg/kg injected three times per week versus 50 µg/kg one time per week in our case) [9], [22]. In sharp contrast, repeated administrations of fluorescent anti-hCD45 mAb did not modify tumor proliferation and the survival of leukemia-bearing mice was not modified. It is consistent with literature where the anti-CD45 mAb is described as a mAb that does not mediate antibody-dependent cellular cytotoxicity or complement-mediated cytotoxicity [23]. Therefore it appears possible to use this anti-hCD45 mAb as a diagnostic tool to detect human leukemia foci in mouse experimental models. Nevertheless, considering that CD45 is a protein tyrosine phosphatase implicated in the regulation of CXCR4 mediated chemotaxis and MAP kinase activation; further tests may be required to document potential impact of the labeled mAb on the sensitivity towards therapeutical agents in oncopharmacology studies. Furthermore, it will be useful to examine any potential impact of anti-hCD45 mAb on disease development in different human leukemia models with varying CD45 expression and determine the imaging sensitivity with low expression levels. These observations could be only performed at the time various human leukemia models will be established.

The FRI performed on mice engrafted with leukemic cells from patient samples showed several leukemic foci in the bones (sternum, tibia, and femur), the liver and the spleen. These results support a previous study performed with several human AML samples [24]. Furthermore, a good correlation between the fluorescent foci detected by the fluorescent anti-hCD45 mAb and the presence of human CD45+ cells (showed by immunohistochemical and flow cytometry analyses) was observed. This demonstrated a specific fixation of the fluorescent mAb on the leukemic foci. The fact that a fluorescent signal can be detected in the liver and the spleen, which have a high absorption coefficient, suggests that the Alexa Fluor 750 was suited to this application.

So, NIR fluorescent anti-hCD45 mAb appears to be an efficient diagnostic tool for human leukemia engraftment in mice but in addition, it could be considered also for the staging of the disease and to assess the efficiency of a therapeutic agent. However for such applications, quantitative fluorescence imaging is required and such a goal can be achieved only using fluorescence tomography and further validation studies.

In conclusion, we demonstrated that the detection of human leukemic foci in mice with a low dose of mAb labeled with a near-infrared fluorochrome is possible but it would be necessary to further determine the limit of *in vivo* detection as compared to reference methods to assess the evolution of the pathology. Of course only CD45+ leukemia cells can be detected with this antibody which is the case of 90% of AML [8].
